# Effects of Apple Polyphenols and Taurine on Growth Performance, Tissue Morphology, and Lipid and Glucose Metabolism in Rice Field Eel (*Monopterus albus*) Fed High Oxidized Fish Oil

**DOI:** 10.1155/2023/4912141

**Published:** 2023-11-29

**Authors:** Shanshan Wu, Jiamin Li, Yao Deng, Peng Fang, Wei Lei, Ao Luo, Zhengwei He, Liufeng Xiong, Gang Yang, Vikas Kumar, Mo Peng

**Affiliations:** ^1^College of Animal Science and Technology, Jiangxi Agricultural University, Nanchang 330045, China; ^2^Department of Fisheries Science, School of Life Science, Nanchang University, Nanchang 330031, China; ^3^Aquaculture Research Institute, Department of Animal, Veterinary and Food Sciences, University of Idaho, Moscow, ID 83844, USA; ^4^Key Laboratory of Featured Hydrobios Nutritional Physiology and Healthy Breeding, Nanchang 330045, China

## Abstract

The aim of this trial was to investigate the effects of apple polyphenols (AP) and taurine (TA) on the growth performance, tissue morphology, and lipid and glucose metabolism in rice field eel fed diets with high oxidized fish oil (OFO). A 10-week feeding experiment was conducted using juveniles (initial body weight 16.66 ± 0.02 g) fed five different diets. Three diets were formulated with various levels of OFO at 9.5, 600, and 800 meq·kg^−1^ and named as Control, POV600, and POV800 diet, respectively. The other two diets were POV600 and POV800 supplemented with 0.5% AP and 0.2% TA, respectively. Compared to the Control group, only the eels fed POV800 exhibited an increase in weight gain and specific growth rate along with a reduction in feed conversion ratio. AP and TA did not affect growth performance; juveniles fed AP, however, showed a decrease in liver weight. Both POV600 and POV800 decreased nuclei number and increased vacuoles size in the liver. POV800 damaged the intestinal structure integrity and reduced goblet cells number. AP repaired the liver damage on nuclei number and vacuoles size in fish fed with POV600 diet, while TA mitigated intestinal histopathological damage on intact structure and goblet cells number. The mRNA expression level of liver *ampkα* in fish fed AP was upregulated, while dietary TA upregulated the mRNA expression levels of liver *ampkα* and *accα*. In the muscle, POV600 downregulated mRNA expression levels of *accα*, *cpt1*, and *lipin*, whereas POV800 upregulated mRNA expression levels of *accα*, *pparα*, and *lipin*. Dietary AP and TA could counteract the effects of POV600 and POV800 diet on muscle lipid metabolism. Both POV600 and POV800 diets upregulated mRNA expression levels of liver *pck1* and *gsk3α*. AP and TA both downregulated mRNA expression level of liver *pck1*, while only TA downregulated the expression of liver *gsk3α*. AP increased the mRNA expression level of *gsk3α* in muscle. In summary, inclusion of AP and TA did not affect growth performance but showed a potential to alleviate liver or intestinal damages induced by a high OFO diet. Dietary AP and TA were also found to regulate mRNA expression of genes related to lipid and glucose metabolism.

## 1. Introduction

Fish oil (FO) plays an essential role in fish growth and lipid metabolism due to its high content in polyunsaturated fatty acids (PUFAs), such as eicosapentaeonic acid (EPA) and docosahexaenoic acid (DHA) [1, 2]. However, these PUFAs are susceptible to oxidation and can become rancid during the FO storage [3]. The resulting peroxides and malondialdehyde (MDA), as the byproducts of oxidized FO (OFO), have been shown to pose toxic side effects on fish [4]. Fish fed the diets containing OFO exhibit abnormal hepatic lipid deposition and oxidative stress and lowered immunity, which in turn reduced growth performance and the overall health of farmed fish. Ultimately, this situation leads to huge losses for the aquaculture [5–7]. Therefore, it is imperative to search for preferable method to reduce the adverse impacts of OFO on aquatic animals. Previous studies have indicated that dietary vitamin C or E can have a beneficial influence in relieving the adverse effect of OFO on red sea bream, black sea bream, and Japanese flounder [8–12]. However, although vitamins are essential and a fundamental feed ingredient, the excessive levels of fat-soluble vitamins such as vitamin E can result in hypervitaminosis-related symptoms [13]. Consequently, there is need to evaluate other functional additives and their effects on growth performance, metabolism, and tissues health in farmed fish fed high OFO diets.

Apple polyphenols (AP) have been reported to enhance antioxidant capabilities [14] and diminish liver lipid deposition [15]. Its impact on growth performance, antioxidant capacity, and lipid metabolism has been investigated in farmed fish. Several studies have reported that a polyphenol-rich diet can improve the growth performance of large yellow croaker (*Larimichthys crocea*) [16] and black carp (*Mylopharyngodon piceus*) [17], elevate the antioxidant capacity of beluga sturgeon (*Huso huso*) [18] and convict cichlid (*Amatitlania nigrofasciata*) [19], and reduce liver lipid deposition of large yellow croaker [16]. Further, dietary supplementation of AP has been found to increase antioxidant capacity and alleviate liver damage in grass carp (*Ctenopharyngodon idella*) [20].

Taurine (TA), on the other hand, has the potential to improve lipid metabolism and alleviate oxidative stress in various fish species, including black carp [21], turbot (*Scophthalmus maximus L*.) [22], European seabass (*Dicentrarchus labrax*) [23], California yellowtail (*Seriola dorsalis*) [24], grass carp [25], and rice field eel (*Monopterus albus*) [26]. The simultaneous effects of these two additives on growth performance and metabolism in farmed fish have not been extensively explored.

AMP-activated protein kinase (AMPK) plays a key role in the regulation of glucose and lipid metabolism in fish [27, 28]. When the intracellular AMP/ATP ratio rises, AMPK becomes activated, leading to the phosphorylation of acetyl-coenzyme A (CoA) carboxylase [29] or peroxisome proliferator-activated receptor *α* (PPAR*α*) [30]. This activation subsequently alleviates the inhibition of carnitine palmitoyltransferase 1 (CPT1) and enhances fatty acid *β*-oxidation. In mammals, it has been demonstrated that AP can reduce liver steatosis induced by high-fat diet via LKB1/AMPK pathway [31]. Similarly, dietary TA has been shown to activate thermogenesis in mouse adipose tissue by regulating the AMPK pathway [32]. However, studies focusing on the effects of dietary AP and TA on the regulation of glucose and lipid metabolism in fish through AMPK pathway and its downstream pathway are still limited.

The rice field eel holds a special economic position in the freshwater aquaculture of China. The fish is known for its burrowing behavior and sensitive to humus-related scents. The formulated feed for this fish often contains a high level of FO. A previous study reported that an OFO diet adversely impacted intestinal function in rice field eel, whereas supplementation with TA was able to restore intestinal function [33]. TA has been shown as an effective additive for enhancing the overall health of this eel species. However, there is still a pressing need to evaluate the effects of dietary TA on tissues function and AMPK pathway in this species fed higher OFO, as well as the potential of including other additives in the diet. Hence, the present study aimed to investigate the effects of TA and AP on growth, liver, and intestinal histology, and lipid and glucose metabolism in rice field eel fed diets with high levels of OFO.

## 2. Materials and Methods

Following the guidelines of the Ethics Committee on Animal Use (CEUA), surgerical methods were performed under anesthesia and all efforts were made to minimize adverse effect on rice field eel.

### 2.1. Experimental Diets

In this experiment, Peruvian steamed fish meal, soybean protein concentrate, and *Saccharomyces cerevisiae* were used as compound protein sources. The carbohydrate component was composed of *α*-starch and flour. Meanwhile, fresh FO and OFO were selected as lipid sources. Fresh FO was oxidized by constant temperature (50°C) in a heated water bath and monitored at 4-hr intervals. The peroxide value (POV) was set based on a previous study of this fish and adjusted as needed [20]. Fresh FO containing a low POV of 9.5 meq·kg^−1^ was used as a lipid source, while the OFO was prepared as another lipid source when the POV reached a high value of 600 meq·kg^−1^ (POV600) and 800 meq·kg^−1^ (POV800). Based on previous studies using rice field eel and grass carp [20, 33], it was very interesting to conduct the following research: evaluating the positive effects of dietary TA in this eel fed POV800 OFO, as well as the potential positive effects of AP in this species fed POV600 OFO. The dietary AP level was set at 5 g kg^−1^ as a supplement for the POV600 OFO diet, and TA was set at 2 g kg^−1^ as a supplement for the POV800 OFO diet. This was done to evaluate the potential positive effects of the two additives in the diet. The five isonitrogenous diets (Table 1) were categorized as follows: Control group (9.5 meq·kg^−1^ fresh FO), POV600 group (POV600 OFO), POV800 group (POV800 OFO), AP group (POV600 diet supplemented with 0.5% AP), and TA (POV800 diet supplemented with 0.2% TA). The feed raw materials were crushed, passed through a 0.25 mm sieve, weighed accurately according to the formula requirements, mixed stepwise, kneaded evenly with FO, and subsequently stored at −20°C.

### 2.2. Experimental Procedure

Rice field eels were cultured in a pond cage at Xihu Farm, Changde, China. The acclimatization method was adopted from a previous study [34]. After acclimatization, fish were starved for 24 hr, and then the uniformly sized and healthy rice field eels were divided into five groups with an average initial weight of 16.66 ± 0.02 g. Each treatment group was divided into three parallel replicates with a total of 15 cages (1.5 m × 2.0 m × 2.0 m). Each cage was stocked with 100 rice field eels. The fish were fed once a day at 17 : 30 to visual apparent satiation. During the 10 weeks experiment, the water temperature was kept at 28 ± 3.5°C, the dissolved oxygen was 6.0 ± 0.5 mg·L^−1^, and the ammonia nitrogen was lower than 0.5 mg·L^−1^.

### 2.3. Sample Collection

At the end of the feeding trial, all fish fasted for 24 hr and then anesthetized with 100 mg·L^−1^ MS-222 based on prior studies [35]. The fish were weighed and counted for each cage. Subsequently, three fish were taken from each cage for body index analysis, wherein the liver and visceral weights of each fish were determined. Liver and muscle from six fish were excised and pooled into 1.5 mL RNase-free tubes, then immediately transferred to liquid N_2_ and stored at −80°C.

### 2.4. Histological Analysis

The liver tip and midgut (5 mm × 5 mm) of the rice field eel were carefully collected and fixed in 4% paraformaldehyde solution for 24 hr, then dehydrated with alcohol, cleaned with xylene, and embedded in paraffin wax. Sections of 5-*μ*m thickness were stained using haematoxylin and eosin (H&E). The method closely followed previous studies, with some necessary adjustments [36, 37]. Histological results of the liver and intestine were observed and photographed by using an Olympus BX53 microscope.

### 2.5. Chemical Composition Analysis

The experimental diets were oven dried at 105°C until constant weight to estimate the moisture content. The Kjeldahl method was used to determine the crude protein content. Crude lipid content was determined using a Soxhlet extraction method. Ash was analyzed after the samples were burned at 550°C in a muffle furnace [38].

### 2.6. Real-Time Polymerase Chain Reaction

Total RNA was extracted from both liver and muscle tissues using Trizol (Invitrogen, USA) and electrophoresed on a 1.2% denaturing agarose gel to test the integrity. Subsequently, the extracted total RNA was reversely transcribed into cDNA using the PrimeScript^TM^ kit (Takara, Japan). Gene sequences were obtained from the National Biotechnology Information Center (NCBI). Primer 5.0 was used to design quantitative primers and verify the amplification efficiency of primers. The primer sequences are shown in Table 2. The qRT-PCR was performed by Mastercycler ep realplex (Eppendorf, Germany), and reaction volume was 20 *μ*L (forward and reverse primers (1 : 1, total 0.8 *μ*L), cDNA (2 *μ*L), SYBR Premix Ex TaqTMII (10 *μ*L), and sterile nonenzyme water (7.2 *μ*L)). The q-PCR program was as follows: one cycle lasted at 95°C for 5 min, followed by 39 cycles of 95°C for 30 s, TM for 30 s (*ampkα1*, *accα*, *pparα*, *pck1*, *gsk3α*, and *ef-1α*: 60.0°C; *cpt1* and *ef-1α*, *lipin* and *β-actin*: 61.0°C), and 72°C for 30 s. The relative expression levels of the target genes were calculated by adopting the comparative Ct method (2^−*ΔΔ*Ct^ method) [39].

### 2.7. Calculations and Statistical Analysis

(1) $$\begin{array}{c} \text{Weight gain}\left(\text{WG},\%\right)=\left(\text{FBW}-\text{IBW}\right)/\text{IBW}\times 100, \end{array}$$

(2) $$\begin{array}{c} \text{Specific growth rate}\left(\text{SGR},\%/d\right)=\left(\text{Ln}\left(\text{FBW}\right)-\text{Ln}\left(\text{IBW}\right)\right)/t\times 100, \end{array}$$

(3) $$\begin{array}{c} \text{Feed conversion ratio }\left(\text{FCR}\right)=\text{FI}/\left(\text{FBW}-\text{IBW}\right), \end{array}$$

(4) $$\begin{array}{c} \text{Condition factor}\left(\text{CF},g/{\text{cm}}^{3}\right)=\text{FBW}/L^{3}\times 100, \end{array}$$

(5) $$\begin{array}{c} \text{Hepatosomatic index}\left(\text{HSI},\%\right)=\text{LW}/\text{FBW}\times 100, \end{array}$$

(6) $$\begin{array}{c} \text{Viserosomatic index}\left(\text{VSI},\%\right)=\text{VW}/\text{FBW}\times 100, \end{array}$$

where FBW is final body weight; IBW is initial body weight; *t* is feeding trial duration in day; FI is feed intake; *L* is body length; LW is liver weight; and VW is visceral weight.

Data statistics and results were expressed as mean values with their standard errors (means ± SEM) and analyzed by SPSS 17.0 (SPSS, IL, USA). For data obtained from two groups, Student's *t*-test was employed to evaluate the significant differences. To evaluate the effect of a high OFO diet, comparisons were made between the Control and POV600 groups, as well as between Control and POV800 groups. Significantly different results were denoted with an asterisk ( ^*∗*^). The effect of dietary AP was evaluated by comparing data between POV600 and AP groups, marked with a hash symbol (^#^). Similarly, the impact of dietary TA was assessed by comparing data between POV800 and TA groups, signified by an ampersand (^&^). The level of significance was set at *P* < 0.05.

## 3. Results

### 3.1. Growth Performance

No significant difference was observed for any of the indices of the Control and POV600 group (*P* > 0.05), as indicated in Table 3. In the POV800 group, the FBW, WG, and SGR were significantly higher compared to those in the Control group, while FCR was significantly reduced (*P* < 0.05). The HSI in AP group was significantly lower than in the POV600 group (*P* < 0.05), but other indices in AP group were comparable to the POV600 group (*P* > 0.05). Notably, all the indices in the POV800 and TA groups did not show significant differences (*P* > 0.05).

### 3.2. Histological Structure in the Liver and Intestine

**Figure 1:**
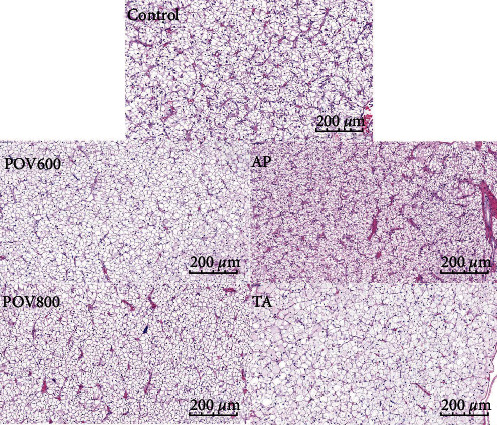
Effect of dietary apple polyphenols (AP) and taurine (TA) on the liver histology. Liver histological characteristics staining with haematoxylin and eosin (H&E) (×40 multiple, *n* = 3). Note: POV600 (600 meq·kg^−1^ OFO diet), POV800 (800 meq·kg^−1^ OFO diet), AP (POV600 group supplemented with 0.5% apple polyphenols), and TA (POV800 group supplemented with 0.2% taurine).

The results of H&E staining of the liver are presented in Figure 1. Compared with the Control group, the two high OFO groups exhibited hepatocytes that were swollen, with a decrease in the nuclei number, and an increase in vacuoles size. Similarly, the liver histology in TA group showed a comparable severe pattern. However, the morphology of hepatocytes in the AP group appeared regular and normal, the number of nuclei increased, and vacuoles size decreased.

**Figure 2:**
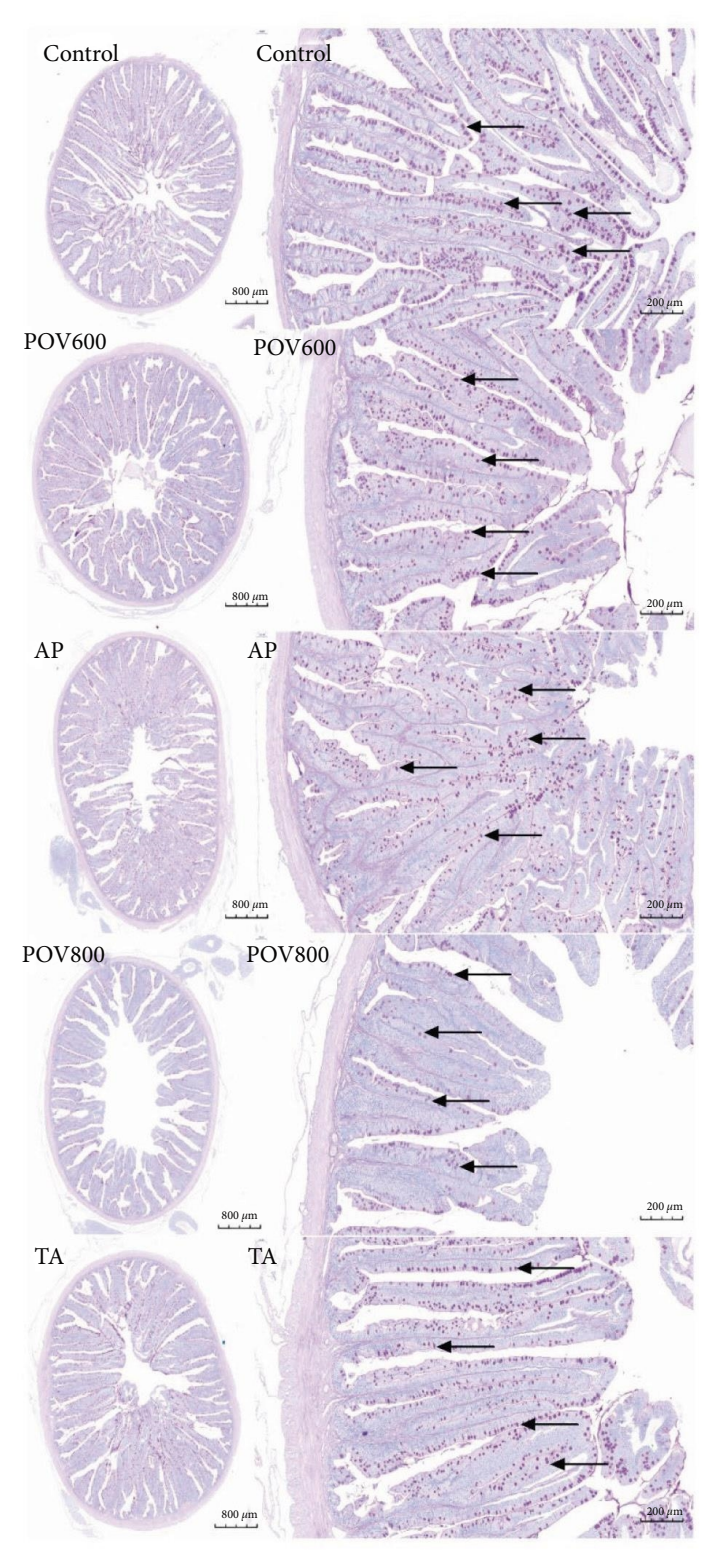
Effect of dietary apple polyphenols (AP) and taurine (TA) on the intestinal histology. Intestinal histological characteristics staining with haematoxylin and eosin (H&E) (×5 and ×20 multiple, respectively, *n* = 3). Note: POV600 (600 meq·kg^−1^ OFO diet), POV800 (800 meq·kg^−1^ OFO diet), AP (POV600 group supplemented with 0.5% apple polyphenols), and TA (POV800 group supplemented with 0.2% taurine).

Subsequent to intestinal H&E staining, as depicted in Figure 2, compared with the Control group, the POV800 group displayed an intestinal structure that was not intact, with a reduction in the number of goblet cells. Supplementing TA in the POV800 group led to an intestinal structure that is more intact along with an increased number of goblet cells. Conversely, the intestinal structure in the POV600 group remained undamaged and with a similar number of goblet cells. Dietary AP supplementation did not yield an improvement in this regard.

### 3.3. Expression of Genes Related to Lipid Catabolism in the Liver and Muscle

**Figure 3:**
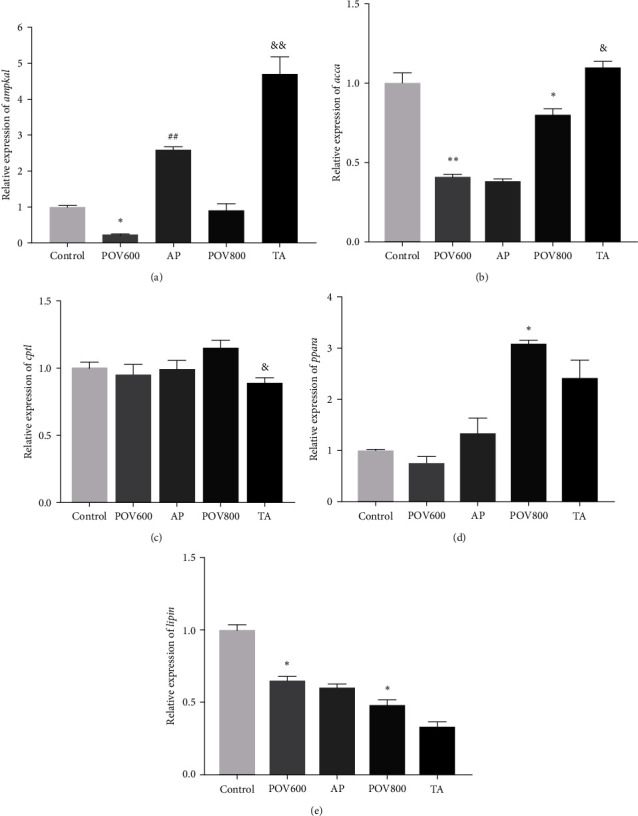
Effects of apple polyphenols (AP) and taurine (TA) on the mRNA expression levels of the liver *ampkα1* (a), *accα* (b), *cpt1* (c), *pparα* (d), and *lipin* (e). Data are expressed as the means ± SEM (*n* = 3).  ^*∗*^ or  ^*∗∗*^ indicates that the POV600 and POV800 groups are significantly different from the Control group at (*P* < 0.05) or (*P* < 0.01), ^##^ indicates that the AP group is significantly different from the POV600 group (*P* < 0.01), and ^&^ or ^&&^ indicates that the TA group is significantly different from the POV800 group (*P* < 0.05) or (*P* < 0.01). Note: *ampkα1 =* AMP-activated protein kinase *α*1; *accα* = phosphorylate of acetyl-coenzyme A (CoA) carboxylase; *cpt1 =* carnitine palmitoyltransferase 1; and *pparα =* peroxisome proliferator-activated receptor *α*. POV600 (600 meq·kg^−1^ OFO diet), POV800 (800 meq·kg^−1^ OFO diet), AP (POV600 group supplemented with 0.5% apple polyphenols), and TA (POV800 group supplemented with 0.2% taurine).

The mRNA expression levels of genes related to lipid catabolism in the liver are presented in Figure 3. Compared to the Control group, the mRNA expression levels of *ampkα1*, *accα*, and *lipin* in the POV600 group, and *accα* and *lipin* in POV800 group, were significantly downregulated (*P* < 0.05 or 0.01). However, the mRNA expression level of *pparα* in the POV800 group was significantly upregulated (*P* < 0.05). In the AP group, there was a significant upregulation in the mRNA expression level of *ampkα1* when compared to POV600 group (*P* < 0.01), while other gene expressions were not significantly affected (*P* > 0.05). Furthermore, the TA group significantly upregulated the mRNA expression levels of *ampkα1* and *accα* when compared to the POV800 group (*P* < 0.05 or 0.01) but significantly downregulated the mRNA expression levels of *cpt1* (*P* < 0.05).

**Figure 4:**
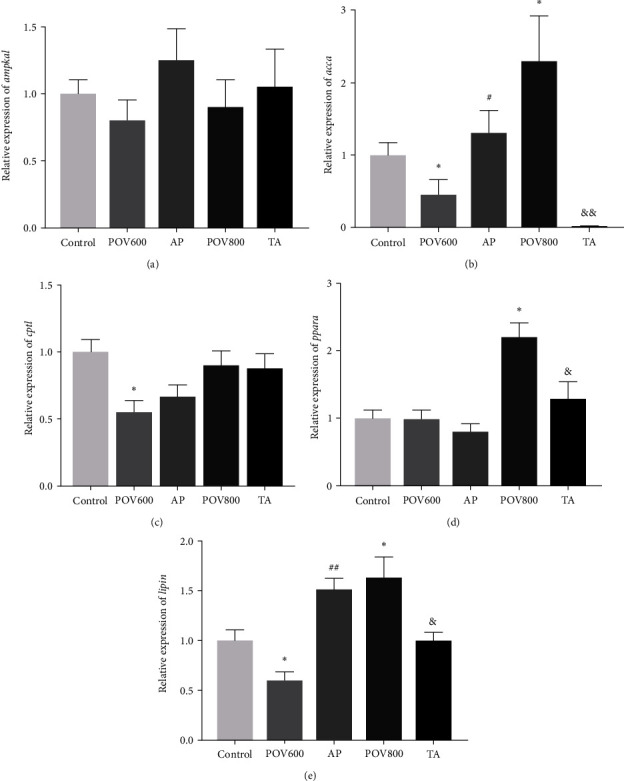
Effects of apple polyphenols (AP) and taurine (TA) on mRNA expression levels of the muscle *ampkα1* (a), *accα* (b), *cpt1* (c), *pparα* (d), and *lipin* (e). Data are expressed as the means ± SEM (*n* = 3).  ^*∗*^ indicates that the POV600 and POV800 groups are significantly different from the Control group at (*P* < 0.05), ^#^ or ^##^ indicates that the AP group is significantly different from the POV600 group (*P* < 0.05) or (*P* < 0.01), and ^&^ or ^&&^ indicates that the TA group is significantly different from the POV800 group (*P* < 0.05) or (*P* < 0.01). Note: *ampkα1* = AMP-activated protein kinase *α*1; *accα =* phosphorylate of acetyl-coenzyme A (CoA) carboxylase; *cpt1* = carnitine palmitoyltransferase 1; and *pparα* = peroxisome proliferator-activated receptor *α*. POV600 (600 meq·kg^−1^ OFO diet), POV800 (800 meq·kg^−1^ OFO diet), AP (POV600 group supplemented with 0.5% apple polyphenols), and TA (POV800 group supplemented with 0.2% taurine).

The expressions of genes related to lipid catabolism in muscle are shown in Figure 4. There was no significant difference observed in the mRNA expression of *ampkα1* among different treatments (*P* > 0.05). The mRNA expression level of *accα* in the POV600 group was significantly lower than those in the Control and AP groups (*P* < 0.05). In the POV800 group, its expression level was significantly lower than those of the Control and TA groups (*P* < 0.05 or 0.01). The mRNA expression level of *cpt1* was downregulated in POV600 group when compared to the Control group (*P* < 0.05). The mRNA expression level of *pparα* in POV800 group was significantly higher compared to those of the Control and TA groups (*P* < 0.05). The mRNA expression level of *lipin* in the POV600 group was significantly downregulated compared to those of the Control and AP groups (*P* < 0.05 or 0.01). In contrast, the expression level of *lipin* in the POV800 group was significantly upregulated compared to those of the Control group and TA group (*P* < 0.05).

### 3.4. Expression of Genes Related to Glucose Metabolism in the Liver and Muscle

**Figure 5:**
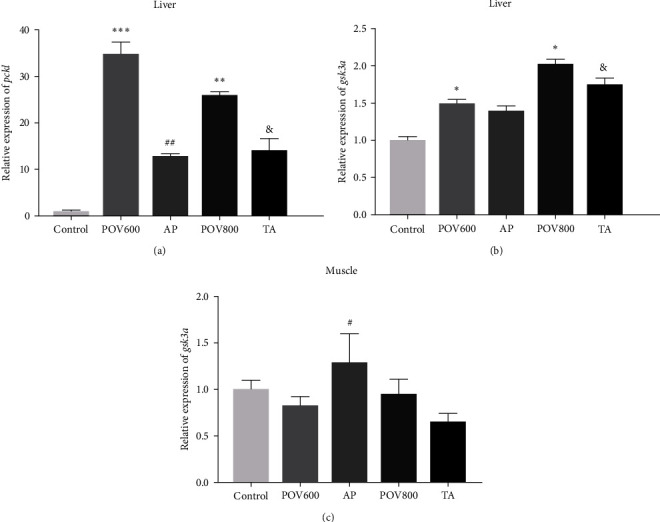
Effects of apple polyphenols (AP) and taurine (TA) on mRNA expression levels of the liver *pck1* (a), *gsk3α* (b), and muscle *gsk3α* (c). Data are expressed as the means ± SEM (*n* = 3).  ^*∗*^,  ^*∗∗*^ or  ^*∗∗∗*^ indicates that the POV600 and POV800 groups are significantly different from the Control group at (*P* < 0.05), (*P* < 0.01) or (*P* < 0.001), ^#^ or ^##^ indicates that the AP group is significantly different from the POV600 group (*P* < 0.05) or (*P* < 0.01), and ^&^ indicates that the TA group is significantly different from the POV800 group (*P* < 0.05). Note: *pck1 =* phosphoenolpyruvate carboxykinase 1 and *gsk3α =* glycogen synthase kinase 3*α*. POV600 (600 meq·kg^−1^ OFO diet), POV800 (800 meq·kg^−1^ OFO diet), AP (POV600 group supplemented with 0.5% apple polyphenols), and TA (POV800 group supplemented with 0.2% taurine).

Compared to the Control group and AP group, the mRNA expression level of *pck1* in liver of the POV600 was significantly upregulated (*P* < 0.01 or 0.001) (Figure 5). The mRNA expression level of *pck1* in liver of the POV800 group was significantly higher compared to those of the Control and TA groups (*P* < 0.05 or 0.01). The mRNA expression level of the hepatic *gsk3α* was upregulated in both POV600 and POV800 groups when compared to the Control group (*P* < 0.05 or 0.01). However, the mRNA expression level of hepatic *gsk3α* in the TA group was downregulated compared to that of the POV800 group (*P* < 0.05).

There was no significant difference in the mRNA expression level of *gsk3α* in the muscle of the Control group and the two POV groups (*P* > 0.05) (Figure 5). The AP group exhibited an upregulated mRNA expression level of *gsk3α* in the muscle when compared to the POV600 group (*P* < 0.05). Moreover, the mRNA expression level of *gsk3α* in the muscle of the POV800 and TA groups showed no significant difference (*P* > 0.05).

## 4. Discussion

After the 10-week feeding trial, the results indicated that feeding an OFO diet resulted in an increased SGR of the rice field eel. This is similar to those observed in largemouth bass (*Micropterus salmoides*) [40]. However, contrasting results have been reported that the addition of OFO to diets led to a reduced SGR in blunt snout bream (*Megalobrama amblycephala*) [41], rohu (*Labeo rohita*) [5], and hybrid grouper (*♀ Epinephelus fuscoguttatus × ♂ Epinephelus lanceolatus*) [42]. These discrepancies might be attributed to nature of the rice field eel as a burrowing animal with a sensitive sense of smell, making it more inclined to feed when exposed to the distinctive scent of OFO [43]. The present study also demonstrated that the FCR of the POV800 group was significantly lower than that of the control group, confirming the earlier predictions. Indices such as CF, VSI, and HSI were not significantly affected by OFO, consistent with the findings in Japanese flounder (*Paralichthys olivaceus*) [10], tilapia (*Oreochromis niloticus*) [44], and amur sturgeon (*Acipenser schrenckii*) [7]. Recent studies have indicated that plant polyphenols and TA may play an important role in improving the health of animals [45, 46]. In studies of tilapia [47] and beluga sturgeon [17], dietary polyphenols contributed to improved growth performance. However, in the current study, the addition of AP to the high OFO diet did not significantly affect the growth of rice field eel, similar to the results from research on grass carp [20]. The difference in growth outcomes might be attributed to fish species or the duration of trial. Notably, TA supplementation did not promote the growth of rice field eel, which contrasts with the positive results observed in turbot [48] and yellowtail kingfish (*Seriola lalandi*) [49]. Interestingly, the addition of AP to a high OFO diet led to a reduction in HSI of rice field eel, which is similar to results observed in sturgeon hybrid of sterlet (*Huso huso ♂ × Acipenser ruthenus ♀*) [50] and Siberian sturgeon (*Acipenser baeri*) [51]. The reason is probably that AP has the capacity to reduce the accumulation of harmful lipids and improve fat metabolism [52].

The histological findings in rice field eel demonstrated that OFO may cause hepatocyte swelling, a reduction in nuclei number, and obvious vacuoles. Similar liver damage caused by OFO has been reported in Chinese mitten crab [6], tilapia [44], and amur sturgeon [7]. In addition, OFO was found to disrupt intestinal structure and decrease the number of goblet cells. This trend of nuclear shift, cytoplasm loss, and incomplete membrane structure was also found in blunt snout bream [41]. During the oxidation process, FO converts into substances like malondialdehyde and other lipid peroxidation products, contributing to the liver and intestinal damage of Amur minnow (*Rhynchocypris lagowskii* Dybowski) [4]. Similarly, OFO caused adverse effect in rainbow trout (*Oncorhynchus mykiss*) [53], rohu [5], and orange spotted grouper (*Epinephelus coioides*) [54]. Thus, it can be speculated that OFO diet has the potential to damage the liver and intestinal histologies of farmed fish. This study indicated that the addition of AP in the high OFO diet was associated with a reduction in the size of liver vacuoles, potentially reflecting a decrease in lipid droplets accumulation. Relative studies in barbel (*Barbus barbus*), maraena whitefish, and Nile tilapia indicated that hepatic vacuoles correlate positively with liver lipid deposition [55–57]. Consistent with other studies conducted in mice and pig [15, 58], dietary AP might serve as an additive to reduce liver lipid deposition. However, dietary AP did not show positive effect on intestinal histology. Furthermore, the effect of dietary TA on intestinal histology was evaluated in this study. Dietary TA supplementation could make the intestinal structure more intact and normal. A previous study reported that dietary TA supplementation restored dysbiosis of intestinal microbiota in rice field eel [33]. Similar findings have been noted in channel catfish (*Ictalurus punctatus*), where TA was found to protect intestinal health by regulating intestinal physical barrier and controlling the inflammatory response at genes expression level [59]. The results of the liver and intestinal histologies suggest that the positive effects of the two additives on liver and intestinal function might vary in this eel.

AMPK pathway serves as a main sensor of cellular energy status, playing a vital role in maintaining energy balance during cell metabolism [60, 61]. Activating AMPK is crucial for promoting lipid metabolism by regulating fatty acid *β*-oxidation [62]. However, whether or not high OFO diet could affect the AMPK pathway, as well as whether dietary AP and TA can influence lipid metabolism in fish through the regulation of AMPK and its downstream pathway, are still not well known. The current study has revealed that a high OFO diet resulted in the downregulation of the mRNA expression levels of *ampkα1* and *accα* in liver, while dietary AP or TA led to upregulation of the mRNA expression levels of *ampkα1* and *accα*. Notably, dietary TA not only upregulated the mRNA levels of *pparα* but also reduced liver lipid content [63]. Comparable findings were reported in studies of groupers [64] and Senegalese sole [65], where dietary TA was linked to better lipid utilization. Therefore, it was hypothesized that dietary TA might reduce liver lipid deposition in rice field eel through the regulation of the AMPK/ACC pathway at the gene level. Specifically, the POV800 diet upregulated the mRNA expression level of *pparα*. A similar effect of an OFO diet on *pparα* was observed in largemouth bass (*Micropterus salmoides*) [66]. Supplementing AP into the OFO diet was associated with the upregulation of the mRNA expression level of *ampkα1* in liver, coupled with downregulation of the mRNA expression level of *cpt1*. Similar effects of dietary AP have been reported in pigs, where it reduced liver triglyceride content and regulated the gene expression related to FA *β*-oxidation in the liver, including *pgc-1α*, *sirt1*, *pparα*, and *cpt1* [15, 67]. This trial is the first to find that dietary AP might activate AMPK and enhance fatty acid *β*-oxidation in fish. However, further robust evidence is necessary to confirm this mechanism.

To comprehensively evaluate the effect of AP and TA on lipid metabolism, various relative indices were measured in the muscle. The results from the present study highlighted intriguing trends: POV600 diets resulted in the downregulation of the mRNA expression levels of *accα*, *cpt1*, and *lipin* in muscle of this fish, whereas POV800 diets led to the upregulation of the mRNA expression levels of *accα*, *pparα*, and *lipin*. Supplementation of AP in POV600 diets and TA in POV800 diets appeared to counteract the inhibition of gene expressions of *accα* and *lipin*, while TA supplementation in POV800 diets decreased the upregulation of *accα*, *pparα*, and *lipin* gene expressions. This suggests that differing high level of OFO diet can yield varying effects on lipid metabolism in the muscle of this eel. In addition, the effect of the two additives within their own OFO level on lipid metabolism was contrary. The effects of the two additives as well as their respective OFO diets on lipid metabolism in the liver and muscle were in contrast to each other. The special patterns of lipid metabolism in this eel deserve further investigation.

Previous studies have consistently indicated that the liver is the main site of glucose metabolism [68, 69]. The OFO diet employed in the study resulted in the upregulation of mRNA expression levels of *pck1* and *gsk3α* in the liver of rice field eel, while no significant effects were observed in the relative gene expression levels in the muscle. These results suggest that OFO diet has the potential to increase liver gluconeogenesis. Supplementation of AP and TA led to the downregulation of mRNA expression levels of *pck1* or *gsk3α*, indicating that both additives would possess the ability to reduce liver gluconeogenesis in this eel. Moreover, it was observed that OFO did not have a noticeable impact on the mRNA expression level of *gsk3α* in the muscle. On the other hand, dietary AP resulted in the upregulation of *gsk3α* expression. Notably, the effects of dietary AP on glycogen synthesis in liver and muscle appeared to differ. This is the first study to delve into the effect of the two additives on glucose metabolism in fish. The specific mechanism of the two additives on glucose metabolism warrants further investigation and discussion.

## 5. Conclusion

This study indicated that high OFO diet exerted adverse effect on the liver and intestinal morphology and lipid and glucose metabolism but not on growth performance. Dietary AP mitigated liver damage, while dietary TA alleviated intestinal damage. Moreover, the two additives regulated lipid and glucose metabolism in liver and muscle, whereas the regulatory metabolic features were different. Therefore, dietary AP and TA can be used as functional additives to counteract the adverse effects of a high OFO diet on health and metabolism of farmed fish.

## Figures and Tables

**Table 1:** Formulation and proximate composition of the diets.

Ingredients	Control	POV600	AP	POV800	TA
Peruvian steam fish meal^a^	40.00	40.00	40.00	40.00	40.00
Soy protein concentrate^a^	20.00	20.00	20.00	20.00	20.00
Beer yeast^a^	5.00	5.00	5.00	5.00	5.00
*α*-Starch^a^	18.00	18.00	18.00	18.00	18.00
Flour^a^	10.96	10.96	10.46	10.96	10.76
Fish oil^a^	3.00	3.00	3.00	3.00	3.00
Premix^b^	1.00	1.00	1.00	1.00	1.00
Monocalcium phosphate^c^	1.20	1.20	1.20	1.20	1.20
Choline chloride^d^	0.80	0.80	0.80	0.80	0.80
Ethoxyquin^e^	0.01	0.01	0.01	0.01	0.01
Calcium propanoate^f^	0.03	0.03	0.03	0.03	0.03
Apple polyphenols^g^			0.50		
Taurine^g^					0.20
Total	100	100	100	100	100
POV (meq·kg^−1^)^h^	9.53	611.48	616.24	816.64	803.57
Proximate analysis (%)					
Crude protein	46.11	45.84	46.19	46.88	46.69
Crude lipid	5.77	6.02	6.08	5.71	5.74
Ash	10.03	9.89	10.16	10.28	10.14

*Note*: POV600 (600 meq·kg^−1^ OFO diet), POV800 (800 meq·kg^−1^ OFO diet), AP (POV600 group supplemented with 0.5% apple polyphenols), and TA (POV800 group supplemented with 0.2% taurine). ^a^These materials were provided by Nanchang Guanglian Industrial Co. Ltd. (Nanchang, Jiangxi, China). ^b^Premix composition (mineral and vitamin, mg/kg diet): KCl 200; KI (1%), 60; CoCl_2_ · 6H_2_O, 50; FeSO_4_·H_2_O, 400; CuSO_4_·5H_2_O, 30; ZnSO_4_·H_2_O, 400; MnSO_4_·H_2_O, 150; Na_2_SeO_3_·5H_2_O (1%), 65; MgSO_4_·H_2_O, 2000; zeolite power, 3645.85; VB1, 12; riboflavin, 12; VB6, 8; VB12, 0.05; VK3, 8; inositol, 100; pantothenic acid, 40; niacin acid, 50; folic acid, 5; biotin, 0.8; VA, 25; VD, 35; VE, 50; VC, 100; ethoxyquin, 150; wheat meal, 2434.15. These materials were provided by MGOTer Bio-Tech Co. Ltd. (Qingdao, Shandong, China). ^c^Monocalcium phosphate was purchased from Chuanheng Ecological Technology Co., Ltd. (Shifang, Sichuan, China). ^d^Choline chloride was purchased from Hebei Dazheng Feed Science & Technology Co., Ltd. (Cangzhou, Hebei, China). ^e^Ethoxyquin was purchased from Weifang Addeasy Bio-Technology Co., Ltd. (Weifang, Shandong, China). ^f^Calcium propanoate was purchased from Shandong Jiutai Bio-Technology Co., Ltd. (Jinan, Shandong, China). ^g^Apple polyphenols (>80%) were purchased from JF-Natural Product R&D CO., Ltd. (Tianjin, China), and Taurine was purchased from Shanghai Aladdin Biochemical Technology Co., Ltd. (Shanghai, China). ^h^The peroxide values (POVs) of the fish oil in the diets of the five groups were 9.5, 600, 600, 800, and 800 meq·kg^−1^, respectively.

**Table 2:** The primers of RT-PCR used in the experiment.

Target gene	Forward sequences (5′–3′)	Reverse sequences (5′–3′)	Size (bp)	Accession number
*ampkα1*	TTGAGTGTGCAGAGGAGGAGGTC	TGAGGTTAGGTGCTGGTCGTCTAG	176	XM_020619494.1
*accα*	GTTCCAACCAAGGCTCCGTATGAC	TCCACAGCAACCACTCCAGTAGG	191	XM_020598745.1
*cpt1*	CCTGGAAGAAGCGTGTCATCAGAC	TGACTGGCAGGTGCTCCTGTATC	168	XM_020625222.1
*pparα*	TTGCCATGCTTGCCTCCAGTATG	GCCATGTCACTGTCGTCCAACTC	175	XM_020621872.1
*lipin*	GCCGCCACCTTGGTTCTGATG	CTGTCCACTCCGCTGTCTCCTC	181	XM_020623677.1
*pck1*	AGACTACACCACCATGCCTCCTG	TTCTCCTCGTCAGAGCCGTCAC	171	XM_020621224.1
*gsk3α*	GTGCCACCGACTACACATCCAAC	TGGTCCACACCGCTGTCTCC	106	XM_020610802.1
*ef-1α*	CGCTGCTGTTTCCTTCGTCC	TTGCGTTCAATCTTCCATCCC	202	XM_020588923.1
*β-actin*	GCGTGACATCAAGGAGAAGC	CTCTGGGCAACGGAACCTCT	156	AY345056

*Note*: *ampkα1 =* AMP-activated protein kinase *α*1; *accα =* phosphorylate of acetyl-coenzyme A (CoA) carboxylase; *cpt1 =* carnitine palmitoyltransferase 1; *pparα* = peroxisome proliferator-activated receptor *α*; *pck1* = phosphoenolpyruvate carboxykinase 1; and *gsk3α* = glycogen synthase kinase 3*α*.

**Table 3:** Effect of dietary apple polyphenols (AP) and taurine (TA) on the growth performance and feed utilization (means ± SEM, *n* = 3).

Index	Control	POV600	AP	POV800	TA
IBW (g)	16.64 ± 0.01	16.65 ± 0.01	16.65 ± 0.01	16.67 ± 0.01	16.67 ± 0.02
FBW (g)	31.74 ± 1.34	34.89 ± 0.27	33.09 ± 0.91	36.60 ± 1.80 ^*∗*^	37.67 ± 1.13
WG (%)	90.75 ± 8.17	109.54 ± 1.77	98.73 ± 5.62	119.54 ± 10.64 ^*∗*^	125.95 ± 6.52
SGR (%/d)	0.92 ± 0.06	1.06 ± 0.01	0.98 ± 0.04	1.12 ± 0.07 ^*∗*^	1.16 ± 0.04
FCR	2.38 ± 0.18	2.06 ± 0.07	2.19 ± 0.09	1.87 ± 0.18 ^*∗*^	1.84 ± 0.08
CF (g cm^−3^)	10.70 ± 0.41	10.07 ± 0.36	10.19 ± 0.37	9.86 ± 0.24	10.59 ± 0.27
VSI (%)	17.31 ± 0.98	15.64 ± 1.03	15.75 ± 0.83	14.50 ± 0.64	16.13 ± 0.67
HSI (%)	6.66 ± 0.27	6.31 ± 0.35	4.39 ± 0.18^#^	6.23 ± 0.21	6.57 ± 0.37

*Note*: IBW = initial body weight; FBW = final body weight; WG = weight gain; SGR = specific growth rate; FCR = feed conversion ratio; CF = condition factor; VSI = visceral somatic index; and HSI = hepatosomatic index. POV600 (600 meq·kg^−1^ OFO diet), POV800 (800 meq·kg^−1^ OFO diet), AP (POV600 group supplemented with 0.5% apple polyphenols), and TA (POV800 group supplemented with 0.2% taurine). In the same row,  ^*∗*^indicates that the POV600 and POV800 groups are significantly different from the Control group and ^#^indicates that the AP group is significantly different from the POV600 group (*P* < 0.05).

## Data Availability

The data are available upon reasonable request to the corresponding author.
